# Using phage to drive selections toward restoring antibiotic sensitivity in *Pseudomonas aeruginosa* via chromosomal deletions

**DOI:** 10.3389/fmicb.2024.1401234

**Published:** 2024-05-15

**Authors:** Jumpei Fujiki, Keisuke Nakamura, Yuko Ishiguro, Hidetomo Iwano

**Affiliations:** ^1^School of Veterinary Medicine, Rakuno Gakuen University, Ebetsu, Japan; ^2^Phage Therapy Institute, Waseda University, Tokyo, Japan

**Keywords:** fluoroquinolones, AMR (antimicrobial resistance), MexXY/OprM, *galU*, phage-resistance, ESKAPE bacteria

## Abstract

Phage therapy has re-emerged in modern medicine as a robust antimicrobial strategy in response to the increasing prevalence of antimicrobial-resistant bacteria. However, bacterial resistance to phages can also arise via a variety of molecular mechanisms. In fact, several clinical studies on phage therapy have reported the occurrence of phage-resistant variants, representing a significant concern for the successful development of phage-based therapies. In this context, the fitness trade-offs between phage and antibiotic resistance have revealed new avenues in the field of phage therapy as a countermeasure against phage resistance. This strategy forces to restore the antibiotic susceptibility of antimicrobial-resistant bacteria as compensation for the development of phage resistance. Here, we present the key achievements of these fitness trade-offs, notably focusing on the enhancement of antibiotic sensitivity through the induction of large chromosomal deletions by bacteriophage infection. We also describe the challenges of this strategy that need to be overcome to promote favorable therapeutic outcomes and discuss future directions. The insights gained from the trade-offs between phage and antibiotic sensitivity will help maximize the potential of phage therapy for the treatment of infectious diseases.

## 1 Introduction

Infectious diseases caused by antimicrobial-resistant bacteria pose significant threats to public health. According to O’Neill (2015), without a global program addressing antimicrobial resistance (AMR), an estimated 10 million people annually could be died to bacterial infections resistant to antibiotics by 2050. Among various types of *Pseudomonas* that commonly inhabit the environment, *Pseudomonas aeruginosa* is the species most frequently associated with infections in humans. This pathogen has the potential to cause various infections including pneumonia (Rossi et al., 2021; Qin et al., 2022). Along with other pathogens like *Enterococcus faecium*, *Staphylococcus aureus*, *Klebsiella pneumoniae*, *Acinetobacter baumannii*, and *Enterobacter* spp., *P. aeruginosa* is classified as an ESKAPE pathogen, which are major contributors to multidrug resistance found in clinical human patients frequently (Mulani et al., 2019). In this context, phage therapy has received significant attention as a promising antimicrobial strategy because phages possess a bactericidal mechanism entirely distinct from antibiotics, enabling them to effectively combat antimicrobial-resistant bacteria (Kortright et al., 2019; Strathdee et al., 2023).

Despite high expectations for phage therapy, previous clinical trials on various phage therapies have reported the emergence of phage-resistant variants (Fujiki et al., 2023; Oromí-Bosch et al., 2023). This represents a significant concern for the successful development of phage-based therapies, similar to the case of resistance seen with classical antibiotics. In the case of *P. aeruginosa*, it has been reported that the polysaccharide O-antigen plays a role in LPS-targeting phages infection. Mutations in the polymerase encoded by *wzy*, responsible for lipopolysaccharide repeat unit polymerization, have been linked to the ability to evade LPS-targeting phages infections. These mutations have been observed in *P. aeruginosa* variants resistant to *Pbunavirus* strains KPP22 (Uchiyama et al., 2016), E215, E217, *Litunavirus* PYO2 and DEV (Forti et al., 2023) and *Pakpunavirus* PaP1 (Li et al., 2018). Moreover, *galU*, for synthesizing the complete core oligosaccharide, plays a crucial role in *Pseudomonas* phage infections. Removing *galU* from the *Pseudomonas* chromosome leads to a deficiency in O-antigen polysaccharide for phage adsorption (Le et al., 2014; Nakamura et al., 2021). Notably, deleting *galU* during phage infection coupled with a substantial chromosomal deletion by Mut associated with the DNA mismatch repair system, results in phage resistance due to the absence of a phage receptor (Shen et al., 2018). Since *hmgA*, responsible for converting red-pigment homogentisic acid to 4-maleylacetoacetate, is located near *galU* on the chromosome, *P. aeruginosa* phage-resistant variants exhibit a brown color phenotype if the extensive chromosomal deletion includes both *galU* and *hmgA*. These bacterial cells are specifically termed as brown mutants (brmts) (Sanz-García et al., 2018; Shen et al., 2018).

Fluoroquinolones, such as ciprofloxacin (CPFX) and levofloxacin (LVFX), are among the most commonly prescribed and effective antimicrobials for treating infections caused by *P. aeruginosa*. Currently, they are the only oral treatment options available for *Pseudomonas* infections (Zakhour et al., 2022). However, *P. aeruginosa* isolated from patients in clinics often exhibits resistance to fluoroquinolones (Rehman et al., 2019; Ruiz, 2019), suggesting that antibiotics commonly used in clinics may no longer be appropriate for this purpose. The main molecular mechanisms through which *P. aeruginosa* can decrease fluoroquinolone sensitivity include mutations in the quinolone resistance determining region (QRDR) in both *gyrA* and *parC*, known to play an integral role in quinolone resistance in *P. aeruginosa* (Rehman et al., 2019). Additionally, it is well-known that *P. aeruginosa* develops resistance through several multi-drug efflux (Mex) systems, such as MexAB-OprM, MexEF-OprN, and MexXY-OprM (Rehman et al., 2019).

To address phage resistance, exploring the fitness trade-offs induced by phage resistance has uncovered new avenues in the field of phage therapy. One strategy aims to restore the antibiotic susceptibility of antimicrobial-resistant bacteria as compensation for the development of phage resistance (Mangalea and Duerkop, 2020; Fujiki et al., 2023; Oromí-Bosch et al., 2023). In a previous study, we found that chromosomal deletions cause simultaneous deletion of the phage receptor encoding *galU* and the drug efflux transporter encoding *mexX* and *mexY* contributing to a trade-off between phage and antibiotics resistance in a *P. aeruginosa* veterinary isolate (Nakamura et al., 2021). However, the applicability of this concept are not completely understood. In this context, we summarize the key achievements related to fitness trade-offs between phage and antibiotics resistance via function of drug efflux transporters. Furthermore, we investigated whether this strategy can be applied to diverse *P. aeruginosa* strains from the aspects of their genome structure, and to quinolone-resistant *P. aeruginosa* strains that are not dependent on drug efflux transporter.

## 2 Subsections

### 2.1 Fitness trade-offs between phage and antibiotics resistance in *Pseudomonas aeruginosa* via drug efflux transporters

**Figure 1:**
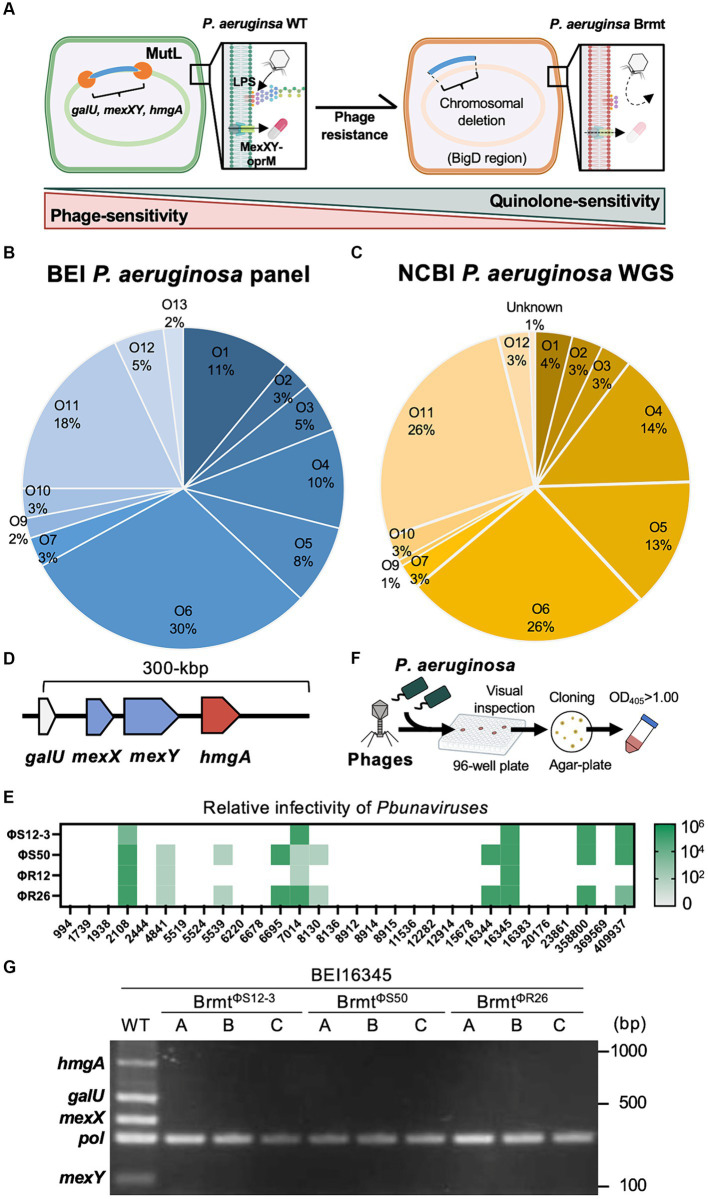
Genomic structures of various *P. aeruginosa* strains surrounding *galU*, and isolation of brmts with QRDR mutations from the *P. aeruginosa* BEI panel. **(A)** Schematic explanation of the trade-offs between phage and fluoroquinolone sensitivity via large chromosomal deletions: once *P. aeruginosa* loses fluctuating sequences containing *galU* and *hmgA*, mutant strains exhibit resistance against LPS-targeting phages and brown pigmentation. It is likely that *mexX* and *mexY* are also simultaneously deleted, resulting in sensitization towards antibiotics that are substrates for the MexXY-OprM system. **(B)** The proportion of O-antigen subtypes in the BEI *P. aeruginosa* clinical panel. **(C)** The proportion of O-antigen subtypes in NCBI GenBank registered *P. aeruginosa* whole-genome sequences (WGS). **(D)** Genomic structure 300-bp upstream of *galU* in 255 strains of *P. aeruginosa*, consisting of 99 strains from the BEI panel and 156 strains from the NCBI-registered sequences. **(E)** Phage sensitivity of *P. aeruginosa* strains with QRDR mutations from the BEI *P. aeruginosa* clinical panel. **(F)** Scheme for brmts selection using the BEI panel and infective *Pseudomonas* phages such as ΦS12-3, ΦS50, ΦR12, and ΦR26. **(G)** Detection of *hmgA*, *galU*, *mexX*, and *mexY* in BEI16345 WT and brmts isolated through the scheme described in **(F)**. *Pol* refers to polymerase for control.

In *P. aeruginosa*, it has been thoroughly investigated that drug efflux transporters themselves cause fitness trade-offs (Mangalea and Duerkop, 2020; Fujiki et al., 2023; Oromí-Bosch et al., 2023). Interestingly, some phages can utilize drug efflux pumps as phage receptors. It was firstly demonstrated by Chan et al. (2016), that ΦOMKO1 is likely to interact directly or indirectly with the outer membrane porin M (OprM), a component of the MexAB and MexXY drug efflux systems. *P. aeruginosa* variants resistant to ΦOMKO1 lack OprM and exhibited increased susceptibility to ciprofloxacin, tetracycline, ceftazidime and erythromycin, which are substrates for Mex system, by up to 50-fold *in vitro*, as well as in infection experiments with *Galleria mellonella* (Gurney et al., 2020). These results strongly indicate that resistance induced by phages targeting drug efflux pumps would be expected to exert evolutionary fitness-cost that alter the function of the efflux pump through genetic mutations or deletions, ultimately leading to reduced antibiotic efflux from the bacteria. In addition to that, it has been reported that chromosomal deletions are involved in drug efflux pump defects in phage-resistant variants. Nakamura et al. (2021) have reported that the *Pseudomonas* virus ΦS12-3, targeting the O-antigen, induces resistance in a *P. aeruginosa* veterinary isolate Pa12 through large chromosomal deletions in the region surrounding *galU* and *hmgA*. These deleted sequences were designated as the Bacteriophages-induced *galU* Deficiency (BigD) region. In that study, *P. aeruginosa* regained sensitivity to quinolones following phage resistance, as the BigD regions contained drug efflux pump encoding genes such as *mexX* and *mexY* (Figure 1A). The results also suggest that LPS-targeting phages can also be used to indirectly induce a fitness trade-off via drug efflux pump function. Recently, Feng et al. (2024) also demonstrated that resistance variants against a LPS-targeting phage exhibit extensive chromosomal deletions, including 290 genes such as *galU, hmgA, mexX*, and *mexY*. This deletion revealed increased susceptibility to aminoglycosides and chlorhexidine, likely due to the loss of *mexX* expression (Feng et al., 2024). On the other hand, large chromosomal deletions containing *mexX* and *mexY* have also been reported to elicit resistance against ΦPIAS which is presumed to infect via the MexY as a receptor (Koderi Valappil et al., 2021). ΦPIAS exhibited synergistic inhibitory effects with MexXY-OprM substrates such as fosfomycin, gentamicin, tetracycline and ceftazidime, on the development of phage-resistant variants compared to phage treatment alone.

### 2.2 The applicability of the concept of antibiotic sensitization via large chromosomal deletions in *Pseudomonas aeruginosa*

To apply the trade-off effect between phage and antibiotic resistance via large chromosomal deletions to diverse strains of *P. aeruginosa*, the gene encoding the MexXY-OprM system must be located at least in the vicinity of *galU* site as demonstrated in the BigD region of the Pa12 isolate (Nakamura et al., 2021). Therefore, in this study, we validated the genomic structure surrounding *galU* in the BEI *P. aeruginosa* clinical isolate panel (100 isolates) (Lebreton et al., 2021) and in NCBI-registered *P. aeruginosa* genomes (156 isolates, Supplementary Table S1). As shown in Figures 1B,C, the composition of O-antigen types exhibited remarkably similar trends in both the BEI clinical isolate panel and the NCBI-registered sequences. Specifically, the O6 antigen comprised the highest proportion at 30% in the BEI clinical isolate panel and 26% in the NCBI-registered sequences, respectively. In addition, one strain among the BEI 100 isolates (BEI13488) was presumed to already possess a large chromosomal deletion due to the brown pigmentation; hence, it was excluded from further analysis. As a result, in all 255 strains, consisting of 99 strains from the BEI panel and 156 strains from the NCBI-registered sequences, it was revealed that within 300 bp upstream of *galU*, both *mexX*, *mexY*, and *hmgA* were present. Particularly, the positioning of *mexX* and mex*Y* was found to be sandwiched between *galU* and *hmgA*, regardless of O-antigen types in all 255 strains. The genomic structure suggests that large chromosomal deletions including *galU* in *P. aeruginosa* are likely to elicit the deletion of *mexX* and *mexY* simultaneously in general. Furthermore, it can be inferred that phage-resistant brmts exhibiting a brown color phenotype due to *hmgA* loss almost certainly have a loss of MexXY; however, further detailed investigation is needed to determine whether the restoration of antibiotics susceptibility occurs due to the loss of MexXY through chromosomal deletions in the variety of phage-resistant variants, because mutations in the QRDR significantly contribute to determining quinolone susceptibility in addition to drug efflux in *P. aeruginosa* (Rehman et al., 2019). Therefore, we selected all 30 strains with mutations in the QRDR from the BEI clinical isolate panel (Lebreton et al., 2021) and attempted to isolate brmts by subjecting these 30 strains to phage infection. Firstly, the infectivity of *Pbunaviruses*, which had been previously isolated (Furusawa et al., 2016; Fujiki et al., 2020), against the 30 strains with QRDR mutations was determined by the presence or absence of plaque formation activity using the efficacy of plating (EoP) method as described elsewhere (Fujiki et al., 2022). As shown in Figure 1E, the host range of these four phages was 16.7% for ΦS12-3, 33.3% for ΦS50, 13.3% for ΦR12, and 33.3% for ΦR26. Using these 10 out of 30 phage-sensitive strains, we attempted to isolate brmts as follows. In brief, each tested *P. aeruginosa* strain at mid-log growth phase (OD_600_ = 0.6) was inoculated into a 96-well plate as shown in Figure 1F, and then infected with the respective phages at an MOI of 0.1. After shaking incubation at 37°C for 24 h, wells showing a red-brown pigments were selected by visual inspection. These wells were then streaked onto LB agar plates, followed by overnight incubation. Subsequently, a single colony was picked for cloning and grown in LB broth overnight. Strains with an OD_405_ of 1.00 or higher were chosen as brmts (Imperi et al., 2009) derived from each clinical isolate. As a result of this selection, brmts were only isolated from strain BEI16345, yielding resistant strains against ΦS12-3, ΦS50, and ΦR26 (Brmt^ΦS12-3^, Brmt^ΦS50^, and Brmt^ΦR26^, three clones selected from each combination: A through C). All brmts exhibited resistance to each phage, as evidenced by the absence of plaques detected in the EoP assay for each respective phage (data not shown). Furthermore, PCR verification of whether each brmt lacked *galU* and key neighboring genes – *hmgA, mexX, mexY* as shown in Figure 1G revealed that all genes were not detected in the phage-resistant brmts compared with BEI16345 parental strain. Finally, the antibiotics susceptibility of these brmts was evaluated using MIC assays. Table 1 shows that there was no restoration in antibiotics susceptibility, particularly for fluoroquinolones (CPFX and LVFX). Thus, the contribution of chromosomal deletions containing drug efflux pumps is partial, indicating that inducing sufficient resensitization to quinolones in strains with mutations in the QRDR via large chromosomal deletions would be difficult.

**Table 1:** MIC values of BEI16345 WT and brmts (μg/mL).

BEI16345	PIPC	IPM	CFPM	CAZ	FOM	LVFX	CPFX	DRPM
WT	>64	0.5	8	>32	<32	>4	>2	<1
Brmt^ΦS12-3^-A	>64	1	8	>32	<32	>4	>2	<1
Brmt^ΦS12-3^-B	>64	1	8	>32	<32	>4	>2	<1
Brmt^ΦS12-3^ -C	>64	1	8	>32	<32	>4	>2	<1
Brmt^ΦS50^-A	>64	1	8	>32	<32	>4	>2	<1
Brmt^ΦS50^-B	>64	1	8	>32	<32	>4	>2	<1
Brmt^ΦS50^-C	>64	1	8	16	<32	>4	>2	<1
Brmt^ΦR26^-A	>64	0.5	8	16	<32	>4	>2	<1
Brmt^ΦR26^-B	>64	0.5	8	16	<32	>4	>2	<1
Brmt^ΦR26^-C	>64	0.5	8	>32	<32	>4	>2	<1

CFPM, cefepime; CAZ, ceftazidime; CPFX, ciprofloxacin; DRPM, doripenem; FOM, fosfomycin; IPM, imipenem; LVFX, levofloxacin; and PIPC, piperacillin.

## 3 Discussion

It has been observed that the function of drug efflux pumps is compromised in phage-resistant variants of *P. aeruginosa* either directly or indirectly due to infection by *Pseudomonas* phages (Chan et al., 2016; Koderi Valappil et al., 2021; Nakamura et al., 2021). Consequently, the restoration of the antibiotics sensitivity has been observed in several studies; however, previous analyses have utilized strains in which the absence of mutations in QRDR was not explicitly confirmed (Chan et al., 2016; Koderi Valappil et al., 2021), or a strain without known QRDR mutations (e.g., Pa12 isolate) (Nakamura et al., 2021). Therefore, it has been suggested for the first time that the loss of MexXY function alone may have limitations in inducing antibiotics sensitization in QRDR mutant strains through the current analysis. In strains harboring mutations in QRDR, it may be possible to induce mutations in DNA gyrase by infecting them with phages that target DNA gyrase within their infection cycle (De Smet et al., 2021), thereby applying another selective pressure. This could potentially impose a fitness cost for antibiotic sensitization through alteration of DNA gyrase function associated with phage resistance.

The acquisition of phage resistance through chromosomal deletions may not be a predominant mechanism for phage resistance. In the selection of brmts as shown in Figure 1F, only one strain out of 30 yielded brmt against four types of phages, suggesting that genetic mutations in phage receptor genes play a significant role in the acquisition of phage resistance. For instance, it has been shown that phage-resistant variants against *Pseudomonas* phages accumulate mutations in the genes such as *wzy* (Uchiyama et al., 2016; Li et al., 2018; Forti et al., 2023), leading to shorter lengths of LPS. However, such mutant strains are characterized solely by point mutations in a single gene and do not involve chromosomal deletions. It has also been reported that no effective antibiotic sensitization was observed in phage-resistant variants without chromosomal deletions in *P. aeruginosa* (Nakamura et al., 2021). Hence, increased selection pressure, leading to higher frequencies of chromosomal deletions involving MexXY, is speculated to be necessary to target the trade-off between phage and antibiotic sensitivity. Shen et al. (2018) reported a positive correlation between the expression level of MutL and the frequency of brmts occurrence in *P. aeruginosa*. Therefore, strategies such as inducing overexpression of MutL or promoting excessive activity of the Mut system in targeted *P. aeruginosa* strains during phage infection might serve as effective selection pressures to enhance the frequency of MexXY loss associated with chromosomal deletions, thereby inducing antibiotic resensitization. Additionally, studies aiming to achieve control of antibiotic sensitivity through the antibiotics themselves has been attempted, and it has been shown that substantial chromosomal deletion strains can also be selected by antibiotic-induced selection pressure (Cabot et al., 2016; Yen and Papin, 2017; Sanz-García et al., 2018). Therefore, there is a potential to impose higher selection pressure effectively by combining phages with other antibiotics. In addition, as shown in Figure 1E, there is also a considerable possibility that phages may not cover *P. aeruginosa* strains targeted. Hence, it is desirable to expand the diverse *Pseudomonas* phages capable of targeting a wide range of *P. aeruginosa* strains prior to actual applications.

To ensure effective management of trade-offs between phage and antibiotic sensitivity in clinical settings, it will be crucial to organize the phages and antibiotics that are involved in such trade-off relationships and understand their molecular basis. In fact, there are many other mechanisms underlying the trade-off between phage and antibiotic susceptibility (Mangalea and Duerkop, 2020; Fujiki et al., 2023; Oromí-Bosch et al., 2023). Interestingly, previous reports (Oechslin et al., 2017; Li et al., 2022; Menon et al., 2022; Wannasrichan et al., 2022) have highlighted other factors contributing to the restoration of antibiotic sensitivity in *P. aeruginosa* strains with large chromosomal deletions associated with phage resistance. These factors include changes in cell membrane potential and alterations in membrane permeability to antibiotics, such as cefepime (Li et al., 2022), fluoroquinolones such as CPFX (Oechslin et al., 2017) and LVFX (Li et al., 2022), and colistin (Menon et al., 2022; Wannasrichan et al., 2022). However, the deleted region in these studies (Oechslin et al., 2017; Li et al., 2022; Wannasrichan et al., 2022) contained *galU* and *hmgA* likely includes *mexX* and *mexY* as demonstrated in this study (Figure 1D). Additionally, the study by Menon et al. (2022) confirmed the presence of *mexX* and *mexY* (as well as *galU* and *hmgA*) within the deleted region. As it has been reported that MexXY contributes to the efflux of cephalosporins (Hocquet et al., 2006) and colistin (Puja et al., 2020), in addition to quinolones, it is important to carefully consider the influence of loss of MexXY as a factor in those antibiotic resensitization associated with phage resistance. Furthermore, another study indicates that antibiotic resensitization is limited when the mutant strain harbors extensive chromosomal deletions but MexXY remains intact (Barceló et al., 2021). Therefore, the contribution of MexXY loss would be considered significant, particularly in strains lacking other resistance mechanisms such as QRDR mutations. On the other hand, our previous analysis of the Pa12 isolate focused on the loss of MexXY associated with chromosomal deletions (Nakamura et al., 2021), but we did not examine changes in membrane potential or other related factors at that time. Therefore, it will be important to carefully examine both aspects and comprehensively discuss the molecular basis of changes in antibiotic sensitivity associated with chromosomal deletions, taking into account factors such as MexXY loss and changes in membrane potential and permeability.

Taken together, this study reveals that *galU* is closely located to *mexX and mexY* in diverse *P. aeruginosa* strains (255 strains), implying that a chromosomal deletion leading to phage resistance is likely to result in the deletion of *mexX and mexY* in general. Based on the previous report (Nakamura et al., 2021), while it could contribute to restoring sensitivity to antibiotics such as fluoroquinolones, the counterexample using the BEI16345 strain has shown that in *P. aeruginosa* strains with mutations in the QRDR, the loss of MexXY due to chromosomal deletions is insufficient to lead to significant changes in sensitivity to fluoroquinolones. This suggests that further studies will be required to apply this kind of trade-off to phage therapy, considering the presence or absence of QRDR mutations.

## Data availability statement

The raw data supporting the conclusions of this article will be made available by the authors, without undue reservation.

## Author contributions

JF: Conceptualization, Data curation, Funding acquisition, Project administration, Supervision, Validation, Visualization, Writing – original draft. KN: Data curation, Validation, Writing – review & editing. YI: Investigation, Writing – review & editing. HI: Funding acquisition, Supervision, Writing – review & editing.
